# Protection and Duration of 23-Valent Pneumococcal Polysaccharide Vaccine Against Hospitalization for Community-Acquired Pneumonia in Older Adults with Low Vaccination Coverage: A Multicenter Matched Case–Control Study in China

**DOI:** 10.3390/vaccines14070646

**Published:** 2026-07-22

**Authors:** Tianchi Yang, Xingqiu Ying, Xiaoqing Wu, Junzhe Shao, Lixia Ye, Yumin Tao

**Affiliations:** 1 Department of Human Resources & Medical Education, Ningbo Municipal Center for Disease Control and Prevention, Ningbo 315010, China; yangtc@zuaa.zju.edu.cn (T.Y.); shaojz@zuaa.zju.edu.cn (J.S.); 2 General Office, Xiaoshan District Center for Disease Control and Prevention (Xiaoshan District Health Supervision Institute), Hangzhou 311203, China; yingxingqiu@zuaa.zju.edu.cn; 3 School of Public Health, Ningbo University, Ningbo 315211, China; 15082864833@163.com; 4 Department of STD and HIV/AIDS Control and Prevention, Ningbo Municipal Center for Disease Control and Prevention, Ningbo 315010, China; 5 Ningbo Kangning Hospital, Ningbo 315010, China

**Keywords:** pneumococcal polysaccharide vaccine, community-acquired pneumonia, hospitalization, case–control, vaccine effectiveness

## Abstract

**Background/Objectives**: 23-valent pneumococcal polysaccharide vaccine (PPV23) effectiveness against community-acquired pneumonia (CAP) remains controversial, with critical gaps in low-coverage settings and beyond 5 years post-vaccination. We estimated real-world PPV23 effectiveness against CAP hospitalization and characterized its duration among elderly adults in a low-coverage region. **Methods**: A multicenter matched case–control study was conducted across 14 hospitals in Eastern China (2018–2022). Cases were patients aged ≥60 years hospitalized with clinically diagnosed CAP. Up to three controls per case were matched on sex, age (±3 years), admission date (±5 days), hospital, and residential community. Conditional logistic regression estimated vaccine effectiveness (VE), adjusting for chronic comorbidities and healthcare utilization. **Results**: Among 6645 cases and 15,806 controls, 5-year PPV23 coverage was 2.14% (cases) and 2.76% (controls). PPV23 was associated with a 22.5% reduction in CAP hospitalization (adjusted VE = 22.5%, 95% CI: 4.1% to 37.3%). Protection was concentrated in non-severe CAP (adjusted VE = 25.7%, 95% CI: 7.0% to 40.7%), with no significant effect in severe CAP (adjusted VE = −11.7%, 95% CI: −115.1% to 42.0%). Extending the exposure window to 6 years yielded no significant VE (adjusted VE = 17.3%, 95% CI: −1.8% to 32.8%). **Conclusions**: PPV23 provides meaningful protection against CAP hospitalization in elderly adults in low-coverage settings, only for non-severe disease. Waning efficacy beyond 5 years supports revaccination at that interval.

## 1. Introduction

Pneumococcal disease continues to represent a significant global public health challenge, with its burden being shouldered disproportionately by older adults [1]. A substantial body of evidence identifies adults aged 65 years and older as a high-risk population for invasive pneumococcal disease (IPD), with case-fatality rates markedly exceeding those observed in younger age groups [2,3,4]. The 23-valent pneumococcal polysaccharide vaccine (PPV23), first licensed in 1983, has been incorporated into national immunization programs in many countries worldwide and is broadly recommended for the prevention of pneumococcal disease among adults aged 65 years and older as well as individuals with underlying chronic conditions [5,6,7]. However, global PPV23 coverage remains suboptimal [8]. Even in high-income European countries, vaccination rates among adults aged 65 years and older remain below 30% [9,10], while coverage in many developing regions, including the Asia-Pacific, is considerably lower [11]. This persistently low uptake, while largely driven by policy and financial barriers, may also be partly rooted in the age-related decline in vaccine responsiveness, which reduces the perceived and actual benefit among older adults.

In China, PPV23 has been available since 2008, and sustained efforts have been made to promote its uptake among older adults at elevated risk. However, vaccination rates have remained persistently low [12]. A 2019 cross-sectional survey across ten provinces reported a coverage rate of only 8.87% among adults aged 65 years and older with chronic diseases [13]. The situation is influenced by a number of interconnected factors. Firstly, with the exception of a few localities, PPV23 is classified as a voluntary, self-paid vaccine in China, with no mandatory provision or public financing. Secondly, pneumonia is not considered a notifiable infectious disease under Chinese law, and clinical etiologic testing is not mandated by regulation. The identification of the causative pathogen in community-acquired pneumonia (CAP) cases remains a challenge [14], compounded by limited access to multiplex PCR and other advanced diagnostic tools [15]. This contributes to a widespread underestimation of the severity of CAP among both the public and healthcare providers [12]. Thirdly, vaccine hesitancy is pervasive [12]: one survey found that, although 37.8% of older adults were aware of PPV23’s preventive benefits, only 1.4% had been vaccinated within the preceding five years [16].

It is important to note that, while the protective efficacy of PPV23 against IPD is well established [7], there has been ongoing academic debate regarding its effectiveness in preventing CAP [17,18]. This uncertainty carries direct implications for cost-effectiveness analyses of PPV23 vaccination programs. Should evidence be found that demonstrates a protective effect against all-cause CAP hospitalization with a high degree of confidence, the cost-effectiveness ratio of PPV23 vaccination would see a substantial improvement [19], thereby strengthening the case for publicly funded immunization strategies.

In light of this, a multicenter matched case–control study was conducted across 14 secondary and tertiary general hospitals in eastern China. The present study sought to rigorously match on hospital, age, sex, date of disease onset, and socioeconomic status (key potential confounders) in an effort to systematically evaluate the protective effectiveness of PPV23 against CAP-related hospitalization in older adults, and to characterize the temporal waning of this protection. The objective of the present study is to furnish concrete evidence for the enhancement of pneumococcal vaccination strategies among the elderly Chinese population within the context of persistently inadequate coverage, a situation that carries significant ramifications for the reduction in the economic burden of pneumococcal pneumonia.

## 2. Materials and Methods

### 2.1. Study Design

We conducted a hospital-based, multicenter matched case–control study to estimate the effectiveness of PPV23 in preventing CAP hospitalization among adults aged 60 years and older, and to characterize how this protection wanes over time since vaccination. The study was carried out across 14 secondary and tertiary general hospitals in Ningbo, a city in eastern China (Table S1). These hospitals include: the First Affiliated Hospital of Ningbo University; Ningbo No. 2 Hospital; Li Huili Hospital of Ningbo Medical Center; Ningbo No. 9 Hospital; the People’s Hospital of Ningbo University; Ningbo Yinzhou No. 2 Hospital; Zhenhai District People’s Hospital of Ningbo; Longsai Hospital of Zhenhai District, Ningbo; Beilun District People’s Hospital of Ningbo; Fenghua District People’s Hospital of Ningbo; Cixi People’s Hospital; Yuyao People’s Hospital; Ninghai First Hospital; and Xiangshan First Hospital. According to the seventh national population census conducted in 2020, the total permanent resident population served by these hospitals was 9,404,300, of whom 1,702,600 (18.1%) were aged 60 years or older. Eligible participants were patients hospitalized at these facilities between 1 January 2018 and 31 December 2022. The study protocol was approved by the Ethics Committee of the Ningbo Center for Disease Control and Prevention (Approval No. 202504) and registered in the Chinese National Medical Research Registration and Information System.

### 2.2. Data Sources

Data were drawn from two linked administrative databases: the hospital discharge diagnosis database and the regional health information platform. Linkage was performed using the residents’ national identification number. The hospital discharge database, coded according to the International Classification of Diseases, Tenth Revision (ICD-10), was used to identify CAP hospitalizations (non-severe CAP: J15.902; severe CAP: J15.903) and to assemble the pool of potential controls. The regional health information platform provided demographic characteristics (age, sex, residential community), vaccination history (PPV23 and influenza vaccine), and comorbidity data (smoking status, alcohol use, body mass index [BMI], hypertension, chronic cardiac or pulmonary disease, diabetes, chronic kidney disease, cancer, and others). For any comorbidity not documented in the clinical record, the individual was assumed to be free of that condition.

### 2.3. Case Definition

Cases were defined as residents of the catchment area aged 60 years or older who were hospitalized for CAP during the study period. CAP was ascertained exclusively from the ICD-10 codes recorded in the hospital discharge database: J15.902 for non-severe CAP and J15.903 for severe CAP.

### 2.4. Control Selection and Matching

Controls were selected from patients hospitalized for cardiovascular diseases at the same participating hospitals. For each case, up to three controls were identified using a nearest-neighbor greedy matching algorithm without replacement, implemented via the MatchIt package (version 4.5.5) in R software. Matching variables included hospital, age (within ±3 years of the case’s date of birth), sex, date of admission (within ±5 days of the case’s admission date), and residential community. Hospital, sex, and residential community were matched exactly; age and admission date were matched within the specified calipers. Post-matching balance was assessed using standardized mean differences (SMDs), with values below 0.10 considered indicative of good balance. Individuals who had been hospitalized or received outpatient treatment for pneumonia (ICD-10 codes: J12–J18) within two months prior to the index date were excluded from the control pool.

### 2.5. Vaccination History

To date, PPV23 (containing capsular polysaccharides of serotypes 1, 2, 3, 4, 5, 6B, 7F, 8, 9N, 9V, 10A, 11A, 12F, 14, 15B, 17F, 18C, 19A, 19F, 20, 22F, 23F, and 33F; no adjuvant) is the only pneumococcal vaccine approved for adults in mainland China, while the pneumococcal glycoprotein-conjugate vaccine is approved only for children. It is recommended for individuals at increased risk of pneumococcal disease, including older adults and those with chronic cardiovascular disease, chronic lung disease, or diabetes. A single dose is routinely given. For individuals with high-risk factors who require a booster dose, an interval of at least 5 years is advised. Vaccination status was ascertained through immunization records in the regional health information platform, which contains dedicated fields for pneumococcal and influenza vaccination. We assumed these records to be sufficiently complete such that the absence of a vaccination entry indicated non-vaccination. PPV23 status was classified as vaccinated if the individual had received PPV23 between 14 days before and 5 years prior to the index hospitalization, and unvaccinated otherwise. To evaluate waning of protection, we prespecified three exposure windows: within 4, 5, and 6 years post-vaccination. Influenza vaccination was defined as receipt of the current season’s influenza vaccine at least 14 days before hospitalization. Neither attending clinicians nor researchers responsible for control selection had access to vaccination records.

### 2.6. Statistical Analysis

Baseline balance between cases and controls was assessed using SMD. An absolute SMD below 0.10 was considered indicative of negligible imbalance; 0.10 to less than 0.20 suggested a minor imbalance; and an SMD of 0.20 or greater indicated substantial imbalance.

Conditional logistic regression was used to estimate matched odds ratios (ORs) and their 95% confidence intervals (CIs) for the association between PPV23 vaccination and CAP hospitalization. Vaccine effectiveness (VE) was calculated as VE = (1 − matched OR) × 100%.

A set of potential confounders was prespecified based on subject-matter knowledge, including demographics (age, sex, residential community), lifestyle factors (BMI category, smoking status, alcohol use), underlying conditions (hypertension, chronic obstructive pulmonary disease, chronic kidney disease, liver cirrhosis, cardiac arrhythmia, diabetes, diabetic complications, and frailty as captured by ICD-10 codes R53 and R54), and medical interventions or preventive measures (antihypertensive therapy, antidiabetic therapy, lipid-lowering therapy, anticoagulant therapy, and influenza vaccination).

Multicollinearity was examined using variance inflation factors (VIFs), with a VIF greater than 10 taken as evidence of severe collinearity. Covariates were retained in the final model if their inclusion or exclusion altered the regression coefficient for the primary exposure (PPV23 vaccination) by more than 10%. In sensitivity analyses, any covariate with a *p* value less than 0.10 for its association with CAP hospitalization was additionally forced into the model. To probe for unmeasured confounding, influenza vaccination—a factor which is not expected to protect against CAP of non-influenza etiology—was treated as a negative control exposure, and its association with CAP hospitalization was estimated via conditional logistic regression. Subgroup analyses were conducted by age group (60–69, 70–79, ≥80 years), sex, and frailty status. Interaction effects across subgroups were evaluated using likelihood ratio tests (LRTs); a *p* value for the interaction term below 0.05 was considered statistically significant.

Sample size was calculated assuming a PPV23 coverage rate of 3% among the elderly, an anticipated VE of 30% [17], a two-sided α of 0.05, and 90% statistical power. Under these assumptions, a minimum of 4536 cases and 13,608 controls (at a 1:3 ratio) was required. The final analytic sample met this requirement.

All analyses were performed using R software (version 4.2.3), with the following packages: tidyverse (v1.3.2), lubridate (v1.9.2), MatchIt (v4.5.5), mice (v3.16.0), Boruta (v9.0.0), glmnet (v4.1.8), and survival (v3.6.4). A two-sided *p* value less than 0.05 was considered statistically significant.

## 3. Results

### 3.1. Basic Characteristics

**Figure 1:**
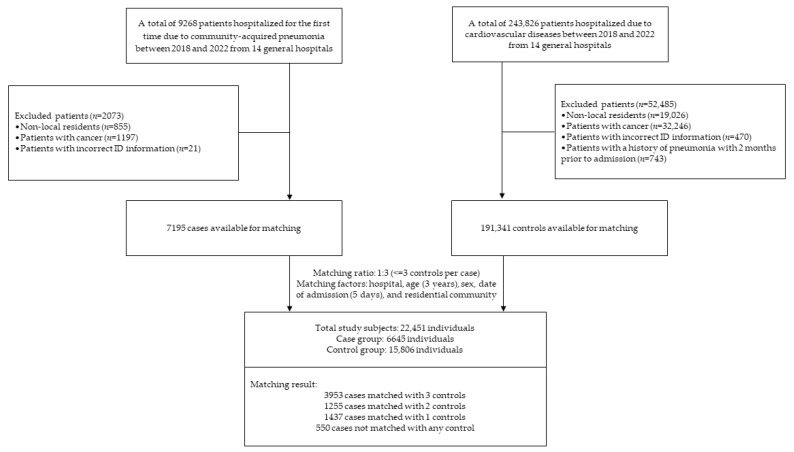
Flow diagram for study enrollment.

Between 1 January 2018 and 31 December 2022, the 14 participating hospitals in the study area admitted a total of 9268 patients aged 60 years or older with CAP. For each case, up to three controls were individually matched based on hospital, age, sex, residential community, and date of admission. Specifically, 3953 cases were matched to three controls each, 1255 to two controls, and 1437 to one control; 550 cases could not be matched to any control. A total of 22,451 individuals—6645 cases and 15,806 controls—were ultimately included in the analysis. The participant selection flowchart is presented in Figure 1.

Among the 6645 CAP cases, 612 were classified as severe pneumonia and 6033 as non-severe pneumonia. The baseline characteristics of cases and controls are summarized in Table 1. The two groups were broadly comparable, with the exception that controls had a higher prevalence of overweight and obesity, as well as higher rates of hypertension, chronic obstructive pulmonary disease, chronic kidney disease, cardiac arrhythmia, diabetes, and diabetic complications. Use of antihypertensive, antidiabetic, lipid-lowering, and anticoagulant medications was also more common among controls.

### 3.2. Vaccine Effectiveness

In the crude analysis, PPV23 vaccination was associated with a 27.4% (95% CI: 10.5%to 41.1%) reduction in the risk of hospitalization for all CAP. The corresponding estimates were 30.4% (95% CI: 13.2% to 44.2%) for non-severe CAP and −6.4% (95% CI: −101.4% to 43.8%) for severe CAP. After adjustment for prespecified covariates, the effectiveness against all CAP hospitalizations was 22.5% (95% CI: 4.1% to 37.3%); for non-severe CAP it was 25.7% (95% CI: 7.0% to 40.7%), and for severe CAP it was −11.7% (95% CI: −115.1% to 42.0%). Detailed results are presented in Table 2.

**Figure 2:**
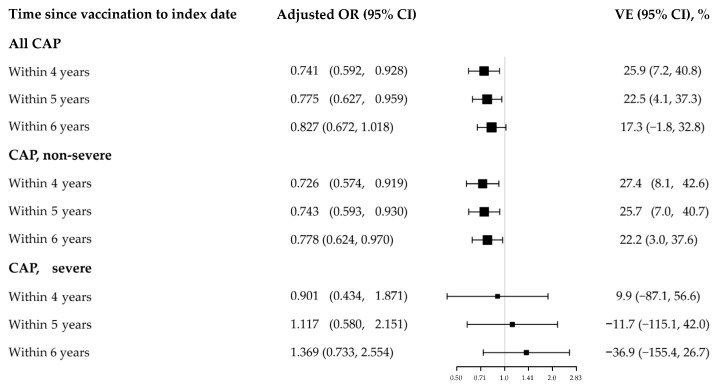
Association of PPV23 vaccination with CAP hospitalization risk by time since vaccination. The size of each square is proportional to the statistical weight (inverse of variance) of the corresponding subgroup, reflecting the relative number of cases within that stratum. Horizontal lines indicate 95% CIs. The gray dashed vertical line indicates the null value (OR = 1.0), which equates to VE = 0%.

We further examined how VE varied by time since vaccination. As shown in Figure 2, a statistically significant protective effect against CAP hospitalization was evident within the first four years after PPV23 receipt (VE = 25.9%; 95% CI: 7.2% to 40.8%). By the sixth year post-vaccination, this protection had attenuated (VE = 17.3%; 95% CI: −1.8% to 32.8%), and the estimate became imprecise. In stratified analyses by pneumonia severity, the protective effect was relatively stable and statistically significant for non-severe CAP, though a declining trend over time was apparent; for severe CAP, no protective effect was observed at any time point.

Subgroup analyses by age group, sex, and frailty status are shown in Table 3. VE was broadly consistent across strata, although point estimates suggested potentially greater benefit among individuals aged 80 years and older, males, and those without frailty.

### 3.3. Sensitivity Analyses

In sensitivity analyses, we refitted the model by simultaneously adjusting for covariates that altered the regression coefficient for PPV23 by more than 10% upon inclusion or exclusion, as well as those with a *p* value less than 0.10 for the outcome in the full model. The results were materially unchanged from the primary analysis (Table S2). Additionally, no significant association was observed between receipt of the seasonal influenza vaccine during the year of hospitalization and CAP hospitalization risk (Table S3), suggesting minimal unmeasured confounding.

## 4. Discussion

Against the backdrop of persistently low vaccination coverage, this multicenter matched case–control study provides a systematic assessment of the protective effect of PPV23 against CAP hospitalization in older adults. The findings indicate that PPV23 confers a statistically significant reduction in the risk of hospitalization for non-severe CAP, whereas no meaningful protection was observed for severe CAP. Moreover, vaccine effectiveness exhibited a clear waning pattern over time since vaccination. By stratifying on disease severity and examining long-term effectiveness in a real-world setting with low coverage, this study yields several actionable insights for pneumococcal vaccination policy in older populations—most notably, confirmation of PPV23’s benefit against non-severe CAP and the need for a booster dose approximately five years after the primary dose.

Our findings are broadly consistent with those of previous studies, though some notable differences warrant discussion. A hospital-based case–control study conducted in Suzhou, Jiangsu Province, China, reported that PPV23 prevented 25.2% of non-severe CAP hospitalizations and 44.0% of severe CAP hospitalizations [20]. However, the estimate for severe CAP hovered at the edge of statistical significance (adjusted OR: 0.56; 95% CI: 0.31 to 1.01; *p* = 0.0548) [20]. Notably, that study selected controls from patients with non-pulmonary respiratory tract infections. Although these patients presented with milder symptoms, their healthcare-seeking behavior for respiratory complaints may have been associated with higher vaccination rates compared to the general source population. This would have inflated the odds of vaccination among controls, thereby underestimating the OR and overestimating the apparent vaccine effectiveness. In contrast, our study used patients hospitalized for cardiovascular diseases as controls, whose vaccination behavior is more likely to reflect that of the true source population. The absence of a protective effect against severe CAP observed in our study may be attributable to several mechanisms. First, patients with severe CAP often have compromised immune function, which may attenuate vaccine-induced protection [21]. Second, serotype replacement with non-vaccine serotypes may diminish the effectiveness of PPV23 in this population [22]. Third, the high prevalence of viral–bacterial co-infection in severe CAP cases may further limit the vaccine’s protective capacity [23,24], as PPV23 targets bacterial pathogens only. Alternatively, the null finding for severe CAP may reflect insufficient statistical power due to the relatively small number of severe cases in our stratified analysis, as evidenced by the wide confidence interval (adjusted OR: 1.117, 95% CI: 0.580 to 2.151).

Globally, the evidence base for PPV23 effectiveness remains markedly heterogeneous [17]. A nested case–control study in the United States that employed urinary antigen testing [25], a multicenter case–control study in Spain [26], and a population-based cohort study [27] have failed to demonstrate a significant protective effect against CAP. Conversely, a 2016 systematic review and meta-analysis of randomized controlled trials [28], a study from Shanghai, China [29], and a matched case–control study from Japan [30] all reported modest but statistically significant protection. A systematic review by Wang et al. similarly concluded that while PPV23 significantly reduced IPD risk, its effectiveness against CAP was inconsistent across studies, underscoring the need for well-designed real-world investigations like ours [31]. This discordance likely stems from differences in study settings (e.g., nursing homes versus community-dwelling populations) [32], age stratification [29,33], circulating serotype dynamics [22], and methodological approaches to case ascertainment and control selection [34].

Beyond the external factors discussed above, the biological underpinnings of vaccine responsiveness in older adults warrant explicit consideration. Immunosenescence—the progressive deterioration of innate and adaptive immunity with age—impairs both the magnitude and functional quality of antibody responses to vaccination [35,36]. This effect is particularly pronounced for polysaccharide antigens like PPV23, which engage B lymphocytes through a T-cell-independent pathway [35]. This fundamental characteristic has two key immunological consequences: first, the response does not involve germinal center formation or generate long-lived plasma cells, and therefore fails to establish immunological memory; second, the lack of T-cell help precludes a true anamnestic response upon revaccination [35,37]. Consequently, functional antibody titers—particularly opsonophagocytic activity—decline progressively [38], typically returning to near-baseline levels within 3 to 5 years post-vaccination [39]. This immunological constraint may not only explain the modest and time-limited effectiveness observed in our study but also contribute to suboptimal vaccine acceptance among older populations as perceived personal benefit wanes. While structural and financial barriers remain primary drivers of low coverage, the intrinsic biological limitation of the vaccine response is an often-underemphasized factor that should inform both communication strategies and future vaccine development.

Conjugate vaccines (PCV13, PCV15, PCV20, and PCV21)—which couple polysaccharides to a carrier protein, generating immunological memory and reducing carriage—have progressively replaced PPV23 as the primary strategy for older adults in high-income countries [40,41,42]. The ACIP now recommends PCV20 monotherapy for all adults aged ≥ 50 years [43], and many European nations have similarly transitioned from PPV23 monotherapy to PCV20 [44,45]. PPV23 nonetheless retains a complementary role, particularly as a follow-on vaccine after PCV15 to extend serotype coverage, given its 23-serotype breadth [42]. In China, however, no adult PCV has been licensed; PCV13 is approved exclusively for children, and PPV23 remains the only available option for older adults for the foreseeable future [46]. While China is rapidly advancing in biomedical research and domestic PCV production for pediatrics [46], the eventual licensure of adult PCVs will likely follow a trajectory similar to that observed in high-income settings. In the interim, our findings of modest, time-limited effectiveness reinforce both the urgency of timely revaccination and the importance of preparing for this eventual transition. An often-overlooked factor is that vaccination coverage itself may modulate individual-level effectiveness estimates through herd immunity or serotype replacement [47]. Studies reporting null findings tend to cluster in high-coverage regions, whereas positive findings are more common in low-coverage settings [18]. In areas with high uptake, herd effects may depress baseline infection risk to the point where incremental individual protection becomes difficult to detect, while serotype replacement can shift the causative pathogen pool toward non-vaccine serotypes [22]. In low-coverage populations, the direct protective effect of vaccination is less obscured by these population-level phenomena and thus more readily observable. Future studies should routinely report local vaccination coverage to facilitate cross-study comparisons.

Regarding duration of protection, our finding of progressively waning effectiveness aligns with a growing body of literature. A meta-analysis of pneumococcal vaccine effectiveness in adults showed that, for CAP requiring hospitalization, VE was substantially lower at five or more years post-vaccination compared with the first five years [17]. A UK retrospective cohort study using electronic health records similarly found that PPV23 effectiveness was approximately 22% in the first year but waned to near zero by year five [48]. Current Chinese guidelines for the management of adult CAP in primary care generally do not recommend revaccination with PPV23 for immunocompetent individuals and stipulate a minimum five-year interval between doses for those who do receive a booster. Our results argue that older adults should be offered a booster dose shortly after the five-year mark following primary vaccination, and that this recommendation should not be restricted to immunocompromised persons alone. This has particular salience in resource-limited settings, where maximizing the return on each vaccine dose is critical.

From an individual patient’s perspective, particularly in a self-paid setting, a 22.5% reduction in the risk of all-cause CAP hospitalization may not appear substantial. However, the cost-effectiveness landscape in mainland China has undergone a marked transformation in recent years. Earlier economic evaluations typically assumed a vaccine price of ¥150–300 per dose based on retail market prices [49]. More recently, however, several major cities (including Shanghai and Guangzhou) have incorporated PPV23 into publicly funded vaccination programs offered at no cost to older residents, and competitive government procurement has driven the unit price down to approximately ¥25 per dose in 2026. This dramatic price reduction fundamentally alters the benefit–cost calculus. A recent cost-effectiveness analysis from Zhejiang Province demonstrated that PPV23 vaccination is highly cost-effective in adults aged ≥ 60 years, with incremental cost-effectiveness ratios of $635.31/QALY and $69.36/QALY for the 60- and 70-year cohorts, respectively—both well below the willingness-to-pay threshold [50]. Thus, while the individual-level protection we observed is modest and wanes over time, the substantially reduced procurement cost under publicly funded programs lends strong support for universal PPV23 vaccination among older adults in China. Public financing not only removes the financial barrier but also reframes vaccination from a discretionary personal expense into a clearly cost-effective societal investment.

Several design features strengthen the validity of our findings. Rigorous individual matching on hospital, age, sex, admission date, and residential community—a proxy for socioeconomic status—combined with standardized mean difference diagnostics to confirm baseline balance, substantially reduced selection bias. The integration of the hospital discharge diagnosis database, which provides more reliable case ascertainment, with the regional health information platform enabled accurate retrieval of vaccination histories and comorbidity data, while linkage via national identification numbers minimized information loss. We also prespecified effectiveness estimates at four, five, and six years post-vaccination, offering a more granular view of waning than most prior studies. Nonetheless, several limitations warrant acknowledgment. First, CAP was identified through ICD-10 codes rather than confirmed by urinary pneumococcal antigen testing or polymerase chain reaction. However, the study was deliberately designed to estimate the effect of PPV23 on all-cause CAP—not pneumococcal CAP of a specific serotype—which mirrors the basis on which public health vaccination decisions are made. Second, although we matched on major confounders and controlled for an extensive set of covariates, residual confounding from unmeasured factors such as health-seeking behavior cannot be entirely excluded. The negative control analysis using influenza vaccination suggested that unmeasured confounding was unlikely to be substantial, though it cannot be ruled out definitively. Third, by restricting enrollment to hospitalized patients, our effectiveness estimates may be conservative relative to the vaccine’s impact on milder outpatient CAP. Future work should incorporate multiplex pathogen detection to disentangle PPV23’s serotype-specific effects from its non-specific benefits and should explore dose–response relationships between population coverage and individual-level effectiveness.

## 5. Conclusions

In summary, this study demonstrates that in a low-coverage setting, PPV23 significantly reduces the risk of non-severe all-cause CAP hospitalization among older adults, albeit with waning protection over time and limited benefit for severe disease. For resource-constrained regions, our findings support the implementation of universal PPV23 vaccination for older adults without the need for elaborate risk stratification, coupled with a booster dose at five years after the primary dose.

## Figures and Tables

**Table 1:** Baseline characteristics of subjects by case–control status.

Characteristics	Cases (*n* = 6645)	Controls (*n* = 15,806)	SMD (95% CI) ^a^
Age (years), mean (S.D.)	72.50 (8.82)	72.87 (8.57)	0.04 (0.01, 0.07)
Age group, n (%)			0.06 (0.03, 0.09)
60–70 years	2827 (42.54)	6313 (39.94)	
70–80 years	2238 (33.68)	5694 (36.02)	
≥80 years	1580 (23.78)	3799 (24.04)	
Sex, n (%)			0.05 (0.02, 0.08)
Female	2692 (40.51)	6020 (38.09)	
Male	3953 (59.49)	9786 (61.91)	
Type of residence, n (%)			0.08 (0.05, 0.11)
Suburban	4199 (63.19)	10,589 (66.99)	
Urban	2446 (36.81)	5217 (33.01)	
BMI category, n (%)			0.11 (0.04, 0.18)
Underweight	810 (65.16)	1703 (64.65)	
Normal	126 (10.14)	195 (7.40)	
Overweight	276 (22.20)	663 (25.17)	
Obese	31 (2.49)	73 (2.77)	
Smoking status, n (%)			0.07 (0.04, 0.11)
No smoke	3255 (73.01)	6869 (69.98)	
Occasionally	4 (0.09)	3 (0.03)	
Often	729 (16.35)	1824 (18.58)	
Have quit smoking	470 (10.54)	1120 (11.41)	
Alcohol drinking status, n (%)			0.04 (0.01, 0.08)
No drink	3399 (76.45)	7582 (77.53)	
Occasionally	216 (4.86)	524 (5.36)	
Often	831 (18.69)	1674 (17.12)	
Underlying disease status, n (%)			
Hypertension	5142 (77.38)	13,043 (82.52)	0.13 (0.10, 0.16)
COPD	1840 (27.69)	5200 (32.90)	0.11 (0.08, 0.14)
CKD	492 (7.40)	1682 (10.64)	0.11 (0.08, 0.14)
Cirrhosis	92 (1.38)	272 (1.72)	0.03 (−0.00, 0.06)
Arrhythmia	942 (14.18)	3191 (20.19)	0.16 (0.13, 0.19)
Diabetes	1495 (22.50)	4422 (27.98)	0.13 (0.10, 0.16)
Diabetes complications	309 (4.65)	1121 (7.09)	0.10 (0.08, 0.13)
Debilitated status	1943 (29.24)	4288 (27.13)	0.05 (0.02, 0.08)
Therapies and preventive measures, n (%)			
Antihypertensive	5034 (75.76)	12,996 (82.22)	0.16 (0.13, 0.19)
Hypoglycemic	2410 (36.27)	6973 (44.12)	0.16 (0.13, 0.19)
Antilipemic	4207 (63.31)	11,758 (74.39)	0.24 (0.21, 0.27)
Anticoagulant	4288 (64.53)	12,103 (76.57)	0.27 (0.24, 0.30)
PPV23 vaccination within 5 years	142 (2.14)	437 (2.76)	0.04 (0.01, 0.07)
Seasonal influenza vaccination	1393 (20.96)	3545 (22.43)	0.04 (0.01, 0.06)

Abbreviations: S.D., standard deviation; BMI, body mass index; COPD, chronic obstructive pulmonary disease; CKD, chronic kidney disease; PPV23, 23-valent pneumococcal polysaccharide vaccine; SMD, standardized mean difference; CI, confidence interval. ^a^ SMD < 0.1 was considered to indicate good balance between the case and control groups.

**Table 2:** Association between PPV23 vaccination and CAP hospitalization risk.

Type of CAP	Vaccinated		Unadjusted OR (95% CI)	Adjusted OR (95% CI) ^a^
	Case, *n* (%)	Control, *n* (%)		
All CAP	142 (2.14)	437 (2.76)	0.726 (0.589, 0.895)	0.775 (0.627, 0.959)
CAP, non-severe	124 (2.06)	396 (2.75)	0.696 (0.558, 0.868)	0.743 (0.593, 0.930)
CAP, severe	18 (2.94)	41 (2.88)	1.064 (0.562, 2.014)	1.117 (0.580, 2.151)

Abbreviations: PPV23, 23-valent pneumococcal polysaccharide vaccine; CAP, community-acquired pneumonia; OR, odds ratio; CI, confidence interval. The reference group was unvaccinated individuals. ^a^ Adjusted for chronic obstructive pulmonary disease and seasonal influenza vaccination.

**Table 3:** Stratified analysis of vaccine effectiveness of PPV23 against CAP hospitalization.

Strata Variables	Vaccinated		Adjusted OR (95% CI) ^a^	VE (95% CI), %	Interaction *p* ^b^
	Case, *n* (%)	Control, *n* (%)			
Age groups					0.996
60–70 years	51 (1.80)	163 (2.58)	0.764 (0.533, 1.095)	23.6 (−9.5, 46.7)	
70–80 years	60 (2.68)	169 (2.97)	0.858 (0.604, 1.216)	14.2 (−21.6, 39.6)	
≥80 years	31 (1.96)	105 (2.76)	0.615 (0.389, 0.972)	38.5 (2.8, 61.1)	
Sex					0.625
Female	55 (2.04)	143 (2.38)	0.828 (0.591, 1.161)	17.2 (−16.1, 40.9)	
Male	87 (2.20)	294 (3.00)	0.746 (0.567, 0.981)	25.4 (1.9, 43.3)	
Status of frailty					0.770
No	90 (1.91)	294 (2.55)	0.613 (0.454, 0.830)	38.7 (17.0, 54.6)	
Yes	52 (2.68)	143 (3.33)	0.630 (0.362, 1.097)	37.0 (−9.7, 63.8)	

Abbreviations: PPV23, 23-valent pneumococcal polysaccharide vaccine; CAP, community-acquired pneumonia; OR, odds ratio; CI, confidence interval; VE, vaccine effectiveness, calculated as (1 − OR) × 100%. The reference group was unvaccinated individuals. ^a^ Adjusted for chronic obstructive pulmonary disease and seasonal influenza vaccination. ^b^ Interaction *p* values were obtained using the likelihood ratio test comparing models with and without the interaction term between PPV23 and each stratification variable. No significant interactions were observed for any stratification variable (*p* > 0.05 for all).

## Data Availability

The data presented in this study is available on request from the corresponding author.

## Supplementary Materials

The following supporting information can be downloaded at: https://www.mdpi.com/article/10.3390/vaccines14070646/s1, Table S1: Geographic distribution, bed capacity, and catchment population of the 14 general hospitals included in the study, Ningbo, China; Table S2: Sensitivity analysis with expanded covariate adjustment: association between PPV23 and CAP hospitalization risk; Table S3: Negative control analysis: association between seasonal influenza vaccination and CAP hospitalization risk.

## Abbreviations

The following abbreviations are used in this manuscript:

PPV23	23-valent pneumococcal polysaccharide vaccine
CAP	community-acquired pneumonia
VE	vaccine effectiveness
IPD	invasive pneumococcal disease
ICD-10	international classification of diseases, tenth revision
SMD	standardized mean difference
OR	odds ratio
CI	confidence interval
