# Supplementary material for: Protection and Duration of 23-Valent Pneumococcal Polysaccharide Vaccine Against Hospitalization for Community-Acquired Pneumonia in Older Adults with Low Vaccination Coverage: A Multicenter Matched Case–Control Study in China
Source: Vaccines (Basel). 2026 Jul 22;14(7):646. doi: 10.3390/vaccines14070646 (PMC13419164; doi:10.3390/vaccines14070646)
Supplement: Supplementary file 1 [file vaccines-14-00646-s001.zip › vaccines-4388041-supplementary.pdf]

Table S1. Geographic distribution, bed capacity, and catchment population of the 14 general hospitals included in the study, Ningbo, China.

No.	Hospital Name	Location (District/County/City)	Beds	Catchment/Service Area	Service Population (10,000)
1	the First Affiliated Hospital of Ningbo University	Haishu District (Yuehu Campus), Fenghua District (Fangqiao Campus), Jiangbei District (Waitan Campus)	4400	Zhejiang Provincial Regional Medical Center	210.77
2	Ningbo No. 2 Hospital	Haishu District	2100	Eastern Zhejiang Region	104.13
3	Li Huili Hospital of Ningbo Medical Center	Yinzhou District	2685	Eastern New Town & National High-tech Zone	160.96
4	Ningbo No. 9 Hospital	Jiangbei District	600	Jiangbei District	48.89
5	the People's Hospital of Ningbo University	Yinzhou District	1650	Yinzhou District & Eastern New Town	160.96
6	Ningbo Yinzhou No. 2 Hospital	Yinzhou District	1150	Yinzhou District	160.96
7	Zhenhai District People's Hospital of Ningbo	Zhenhai District	600	Zhenhai District	51.05
8	Longsai Hospital of Zhenhai District, Ningbo	Zhenhai District	320	Zhenhai District	51.05
9	Beilun District People's Hospital of Ningbo	Beilun District	800	Beilun District	82.94
10	Fenghua District People's Hospital of Ningbo	Fenghua District	720	Fenghua District	57.75
11	Cixi People's Hospital	Cixi City	1035	Cixi City	182.95
12	Yuyao People's Hospital	Yuyao City	1199	Yuyao City	125.40
13	Ninghai First Hospital	Ninghai County	850	Ninghai County	69.60
14	Xiangshan First Hospital	Xiangshan County	1000	Xiangshan County	56.77

Table S2. Sensitivity analysis with expanded covariate adjustment: association between PPV23 and CAP hospitalization risk.

Type of CAP	PPV23 vaccination status		Adjusted OR (95% CI) [a]	VE (95% CI), %
	Case, *n* (%)	Control, *n* (%)		
All CAP	142 (2.14)	437 (2.76)	0.748 (0.603, 0.928)	25.2 (7.2, 39.7)
CAP, non-severe	124 (2.06)	396 (2.75)	0.710 (0.565, 0.893)	29.0 (10.7, 43.5)
CAP, severe	18 (2.94)	41 (2.88)	1.176 (0.608, 2.272)	-17.6 (-127.2, 39.2)

Abbreviations: PPV23, 23-valent pneumococcal polysaccharide vaccine; CAP, community-acquired pneumonia; OR, odds ratio; CI, confidence interval; VE, vaccine effectiveness, calculated as (1-OR)×100%.

The reference group was unvaccinated individuals.

[a] Adjusted for BMI category, smoking status, hypertension, chronic obstructive pulmonary disease, diabetes, diabetes complications, chronic kidney disease, liver cirrhosis, cardiac arrhythmia, frailty, antihypertensive therapy, antidiabetic therapy, lipid-lowering therapy, anticoagulant therapy, and seasonal influenza vaccination.

Table S3. Negative control analysis: association between seasonal influenza vaccination and CAP hospitalization risk

Type of CAP	Influenza vaccination status		Adjusted OR (95% CI) [a]	VE (95% CI), %
	Case, *n* (%)	Control, *n* (%)		
All CAP	1393 (20.96)	3545 (22.43)	1.023 (0.938, 1.117)	-2.3 (-11.7, 6.2)
CAP, non-severe	1226 (20.32)	3141 (21.84)	1.023 (0.933, 1.122)	-2.3 (-12.2, 6.7)
CAP, severe	167 (27.29)	404 (28.41)	1.020 (0.784, 1.328)	-2.0 (-32.8, 21.6)

Abbreviations: CAP, community-acquired pneumonia; OR, odds ratio; CI, confidence interval; VE, vaccine effectiveness, calculated as (1-OR) × 100%.

The reference group was unvaccinated individuals.

[a] Adjusted for chronic obstructive pulmonary disease and 23-valent pneumococcal polysaccharide vaccination.
